# Phylogenetic Reconstruction of Orthology, Paralogy, and Conserved Synteny for Dog and Human

**DOI:** 10.1371/journal.pcbi.0020133

**Published:** 2006-09-29

**Authors:** Leo Goodstadt, Chris P Ponting

**Affiliations:** Medical Research Council Functional Genetics Unit, University of Oxford, Department of Physiology, Anatomy, and Genetics, Oxford, United Kingdom; Baylor College of Medicine, United States of America

## Abstract

Accurate predictions of orthology and paralogy relationships are necessary to infer human molecular function from experiments in model organisms. Previous genome-scale approaches to predicting these relationships have been limited by their use of protein similarity and their failure to take into account multiple splicing events and gene prediction errors. We have developed PhyOP, a new phylogenetic orthology prediction pipeline based on synonymous rate estimates, which accurately predicts orthology and paralogy relationships for transcripts, genes, exons, or genomic segments between closely related genomes. We were able to identify orthologue relationships to human genes for 93% of all dog genes from Ensembl. Among 1:1 orthologues, the alignments covered a median of 97.4% of protein sequences, and 92% of orthologues shared essentially identical gene structures. PhyOP accurately recapitulated genomic maps of conserved synteny. Benchmarking against predictions from Ensembl and Inparanoid showed that PhyOP is more accurate, especially in its predictions of paralogy. Nearly half (46%) of PhyOP paralogy predictions are unique. Using PhyOP to investigate orthologues and paralogues in the human and dog genomes, we found that the human assembly contains 3-fold more gene duplications than the dog. Species-specific duplicate genes, or “in-paralogues,” are generally shorter and have fewer exons than 1:1 orthologues, which is consistent with selective constraints and mutation biases based on the sizes of duplicated genes. In-paralogues have experienced elevated amino acid and synonymous nucleotide substitution rates. Duplicates possess similar biological functions for either the dog or human lineages. Having accounted for 2,954 likely pseudogenes and gene fragments, and after separating 346 erroneously merged genes, we estimated that the human genome encodes a minimum of 19,700 protein-coding genes, similar to the gene count of nematode worms. PhyOP is a fast and robust approach to orthology prediction that will be applicable to whole genomes from multiple closely related species. PhyOP will be particularly useful in predicting orthology for mammalian genomes that have been incompletely sequenced, and for large families of rapidly duplicating genes.

## Synopsis

Biologists often exploit the evolutionary relationships between proteins in order to explain how their findings are relevant to the biology of other species, including *Homo sapiens.* The most natural way to define these relationships is to draw family trees showing, for example, which human protein is the counterpart (“orthologue”) of a protein in dog, and which human proteins have arisen by recent duplication of existing genes (“paralogues”). On a small-scale this is relatively straightforward, but it is difficult to do this automatically on a genome-wide scale. In this paper the authors describe a new approach to drawing a giant family tree of all proteins from humans and dogs. They show how this tree allows them to refine some protein predictions and discard others that are likely to be nonfunctional dead sequences. Family relationships can show how the dog and human genomes have been rearranged since their last common ancestor. In addition, they help to identify the proteins that are specific to either dog or human, and which contribute to these species' biological differences. Giant trees, drawn from this method, will help to associate the differences, duplications, and evolution of proteins in different mammals with their distinctive physiologies and behaviours.

## Introduction

Distinguishing orthologues (genes that arose via a speciation event) from paralogues (genes that arose via duplication within a genome) is critical to comparative biology. This is because orthology is the basis by which molecular function in humans can best be inferred from experimental results in model organisms. Orthologous genes are descended from a single gene in the last common ancestor of their two species [1]. They are hence most likely to share a conserved ancestral gene function.

**Figure 1:**
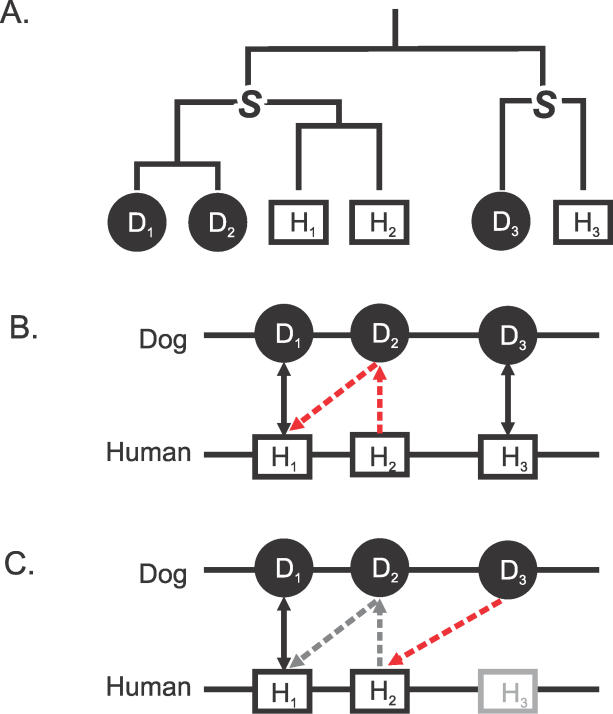
The Assignment of Orthology by Ensembl (A) Shows the true phylogenetic relationships for three dog (D_1–3_) and three human gene homologues (H_1–3_). D_3_ and H_3_ are 1:1 orthologues, having being derived from a single gene at the last common ancestor (marked “S” for speciation point). D_1_, D_2_ and H_1_, H_2_ are likewise orthologues of each other but in a many-to-many relationship. (B) Shows that D_1_ and H_1_ and D_3_ and H_3_ are BLAST reciprocal best hits (solid arrows; “UBRH” in Ensembl terminology). Because the D_2_ and H_2_ loci are closely linked neighbours of the H_1_ loci, their orthology relationships are also predicted by Ensembl on the basis of their BLAST nonreciprocal best hits: H_1_ is the best hit for D_2_, and D_2_ is the best hit in turn for H_2_ (dashed red arrows; “RHS” in Ensembl terminology). Because of this lack of reciprocity, H_1_ is simultaneously in a many-to-one relationship with D_2_ (and H_2_) and a one-to-many relationship with D_1_ and D_2_. As orthology is, by definition, a transitive property between genes of two species, this inconsistency can be reconciled by linking all four genes together into a single set of orthologues, in effect adding the missing link between D_1_ and H_2_. Many such inconsistencies can be found in version 27.1 of the Ensembl Compara database, for example, ENSCAFG00000009718, ENSCAFG00000009724, ENSG00000180305, and ENSG00000182931 are found in relationships illustrated by D_1_, D_2_, H_1_, and H_2_, respectively. (C) Human gene H_3_ has not been predicted. The highest-scoring BLAST alignment for its orphaned orthologue D_3_ becomes H_2_ (dashed red arrow). This erroneous assignment of orthology for D_3_ arises because Ensembl does not distinguish between adjacent in-paralogues such as H_1_ and H_2_, and out-paralogues such as H_3_.

Genes may be duplicated to give rise to multiple additional copies, often lying in tandem. Lineage-specific duplicates (“in-paralogues” [2]) from two species together form an “orthologous clade” whose members are all descended from a single ancestral gene in the last common ancestral species (Figure 1). The functions of in-paralogues can be used to infer species-specific biology [3]. Analyses of the genome sequences of human, mouse, rat, and chicken genomes [4–7] reveal that tandemly duplicated genes in vertebrates are overrepresented in four broad functional categories: chemosensation, reproduction, immunity and host defence, and toxin metabolism. These reflect common themes in within-species (such as for mate selection) and interspecific (such as for resistance to infection) competition [3]. The correct differentiation of paralogues from orthologues is thus crucial if their biological significance is to be appreciated fully.

Traditionally, orthology relationships for individual gene families have been predicted by carefully constructed multiple alignments and by reconstructing phylogeny via the use of either maximum likelihood [8] or parsimony [9] methods. However, for genome-scale investigations, current methods do not yet automatically generate multiple alignments of unfailing quality, especially in the face of variable genomic data and gene prediction quality, rendering subsequent phylogenetic steps unreliable. Instead, orthology across whole genomes has been determined automatically using reciprocal best hits in all-against-all comparisons of amino acid sequences [2,6,7,10]. Two sequences are identified as orthologous if they find each other as the highest scoring alignments among all sequences from the other species.

This procedure is most reliable for relatively flawless datasets, such as those from prokaryotes, but works less well where gene sets are incomplete and the predictions imperfect. In vertebrate eukaryotic species, the gene sets used for orthology prediction are much more likely to contain errors simply because the challenges for gene prediction are so much greater. Vertebrate genomes employ long introns, alternative splicing, and cryptic splice sites, and are more likely to contain sequencing errors, such as base changes and insertion–deletions, or assembly errors causing inversions and translocations. As a result, exons and transcripts may be absent from predicted genes, and more rarely, pseudogenes may be predicted wrongly as genes.

When duplications have occurred in one or both lineages, the resulting orthologous genes are in one-to-many or many-to-many relationships (Figure 1A), respectively. For each set of orthologues, relying solely on reciprocal best hits will, by definition, only identify one pair out of all orthology relationships. The remaining in-paralogues need then to be determined, in a second step, by adding genes that have high scoring alignments with this initial orthologue pair.

Ensembl [11] and Inparanoid [2] are widely used methods for predicting orthology and paralogy. Both approaches start with reciprocal best-hitting protein sequence pairs. Each method assumes that genes are best represented by the longest transcript, and no other splice variants are considered. For wholly sequenced, closely related genomes, Ensembl takes advantage of the observation that most in-paralogues are generated by tandem duplications. This tends to preserve gene order. Accordingly, if there is a series of orthologues defined by BLASTP [12] reciprocal best-hitting pairs that have the same relative gene order in both species and which fall within a tuneable genomic window size (e.g., 1.5 Mbp [13]), and if protein sequences from the intervening genes have high scoring BLAST hits to the initial orthologues, then these too will be gathered into an orthologue set [14]. Because this is not a reciprocal operation, some of the resulting orthologues between two species inevitably exhibit contradictory, nontransitive relationships (Figure 1B): a gene may be identified as belonging to a “one-to-many” set with respect to one species (suggesting gene duplication only in species one), but then also as part of a “many-to-one” set with respect to another (gene duplication only in species two) [6].

This Ensembl process does not correctly distinguish, in many cases, between “in-paralogues” (lineage-specific duplicates) and “out-paralogues” (duplicated genes present in the common ancestor of the two species). Where there have been lineage-specific gene losses or failures in gene prediction, then the corresponding gene in the other species should be identified as an “orphan” (an unpaired gene). Instead, Ensembl may assign such orphaned genes wrongly as members of a neighbouring orthologue family, even if they are distant homologues (Figure 1C). In effect, Ensembl assumes that lineage-specific gene losses or absences occur rarely. A number of mammalian genomes are being sequenced at low statistical coverage (~2-fold, whole-genome shotgun) [15] and will be therefore highly fragmentary and incomplete. This results in large numbers of missing genes and a loss of independent synteny information, both of which will require Ensembl to modify their current approach to finding paralogues in closely related genomes.

Inparanoid employs stricter criteria for assigning paralogues than Ensembl [2]. Again, the main orthologue pair of each set of orthologues is first identified as the reciprocal best pairwise match. Unlike Ensembl, additional orthologues are then added to this set only if their proteins are more sequence-similar to the initial orthologue from the same species. By design, and partly for reasons of computational cost, Inparanoid examines only pairwise relationships and thus does not construct phylogenies. Instead, the method has a careful set of heuristics to merge, delete, or separate predicted orthologue sets with overlaps. These heuristics can only be readily understood given an implicit underlying phylogenetic model.

Both Inparanoid and Ensembl labour under three limitations. First, orthology and paralogy relationships are properties of the evolutionary history of a gene family [1], barring partial gene conversions. Phylogenetic trees are thus the most natural way to represent the familial relationships among homologues. Relying solely on pairwise relationships fails to make optimal use of all available information.

Second, underlying assumptions for both methods are that protein similarity accurately reflects evolutionary distance and that paralogues evolve at equal rates [2]. For recently duplicated genes, many of which are or have been evolving adaptively, the reliance on uniform selection upon all members of a gene family is particularly treacherous. Even in the general case, the rate of amino acid substitution varies by up to 300-fold [16]. As a result, orthologue sets may contain a disproportionate number of large families that have ancient divergences but are highly conserved. Rapidly evolving genes with recent provenances will be underrepresented.

Third, Ensembl and Inparanoid make no explicit provision for handling genes with multiple transcripts. Both describe orthology and paralogy in terms of genes, and yet assign orthology not directly from gene comparisons but indirectly via protein sequence comparisons. However, where there are alternatively spliced variants, there is no obvious way to chose between all the possible sequence comparisons involving different variants. Ensembl, and analyses using Inparanoid, skirt around this problem by discarding all but the longest transcripts. However, there is no guarantee that the longest transcripts from orthologous genes are themselves orthologous throughout because they may each employ different combinations of exons.

We consequently sought a new approach to predicting orthology and paralogy relationships which: (1) would be applicable to large-scale analyses of multiple entire genomes; (2) directly produces phylogeny; (3) would be less susceptible to variations in evolutionary rates; (4) handles multiple transcripts explicitly; and (5) would not rely on synteny information. PhyOP (phylogenetic orthology and paralogy) has been designed to meet all of these requirements.

Unlike Inparanoid and Ensembl, PhyOP explicitly reconstructs phylogenies of transcripts to take advantage of all available sequence data. Gene orthology predictions are made by comparing the transcript phylogeny with the known species tree. PhyOP predicts orthology using a distance metric based not on amino acid substitutions, as in all other approaches to large-scale orthology prediction, but rather on *d_S_,* the number of synonymous nucleotide substitutions per synonymous site. Because silent mutations in coding DNA sequences do not lead to changes in the protein products, synonymous sites are under fewer evolutionary constraints than other coding sites [17], and hence more accurately reflect underlying neutral rates and the true evolutionary distance between genes [18]. *d_S_* values vary only by approximately 2-fold or 3-fold within mammalian genomes [6,19,20], which is two orders of magnitude lower than variations in the amino acid substitution rate [16].

Over long evolutionary distances, however, the method becomes increasingly less appropriate because of saturation at synonymous sites. Nevertheless, aggregate *d_S_* estimations have been employed even for relatively divergent species pairs, such as human and chicken, which are separated by more than 300 million years [4].

Our approach also differs from methods that rely on conserved gene order in inferring orthology. The use of PhyOP is thus appropriate both for relatively complete and for incomplete genomes such as those assembled only into short contigs. Having achieved this aim, we realised that we could exploit conserved gene order information so as to determine the method's efficacy.

We use, as our basis dataset, Ensembl [21] genes for the newly sequenced dog *(Canis familiaris)* genome sequence together with the corresponding set for human *(Homo sapiens).* This provided an opportunity to compare the degrees of lineage-specific gene duplication in dog and human, and to consider the fraction of single orthologues that have persisted in both lineages, without apparent loss or duplication, since their common ancestor.

Dog and human orthologues predicted by PhyOP can be downloaded from http://wwwfgu.anat.ox.ac.uk:8080/phyop_orthologs, and the software implementation is freely available from the authors.

## Results

PhyOP is a phylogenetic method that uses the synonymous substitution rate *d_S_* as a proxy for the evolutionary distance. Currently, it is only suitable for the accurate prediction of orthology among species of recent divergence, such as the mammals. To recover gene phylogeny and to predict orthology, the design of PhyOP had to overcome five challenges. 1) Like other metrics relying on nucleotide sequence, *d_S_* saturates over large evolutionary distances and cannot be used to distinguish gene duplications that are more ancient than the divergence of the first mammals. Phylogenetic algorithms were required to be modified in order to disregard saturated values at large evolutionary distances (see Materials and Methods). 2) Inconsistent gene phylogenies due to missing or erroneous transcript predictions had to be resolved. 3) We also had to resolve apparent cases of merged genes in the Ensembl gene set, where adjacent and separate paralogues have apparently been amalgamated erroneously into a single prediction. 4) Pseudogenes present in the Ensembl gene set had to be distinguished from functional genes. Missing or inserted bases in high-coverage genome sequence are rare (estimated to be fewer than one in 10^4^: see, for example, [22–24]). Yet even these would interrupt the reading frames of approximately 4% of protein coding genes (assuming 350 codons per gene). Apparent disruptions in otherwise functional genes can also result from missing exons and other problems in predicting exon boundaries, as well as from insertion–deletion single nucleotide polymorphisms. Ensembl attempts to rescue these functional genes by introducing short (≤ 10 bp) artificial introns around disruptions to the reading frame. However, as it is impossible to know a priori which disruptions are artefactual, the Ensembl procedure carries the necessary cost of mispredicting some nonfunctional pseudogenes with real in-frame disruptions as functional genes. 5) Optimal reconstruction of gene orthology and paralogy required phylogenies derived from all, rather than a subset of, transcripts.

### Transcript Phylogeny

Other orthology prediction methods assume that the longest transcripts of orthologous genes would themselves be orthologous. Instead of making such an a priori choice of transcripts, we realised that a phylogeny of all available transcripts from both species would necessarily disambiguate transcripts that are orthologous from those that are paralogous. This could then be used as a secure basis from which to infer phylogeny on the gene level.

**Figure 2:**
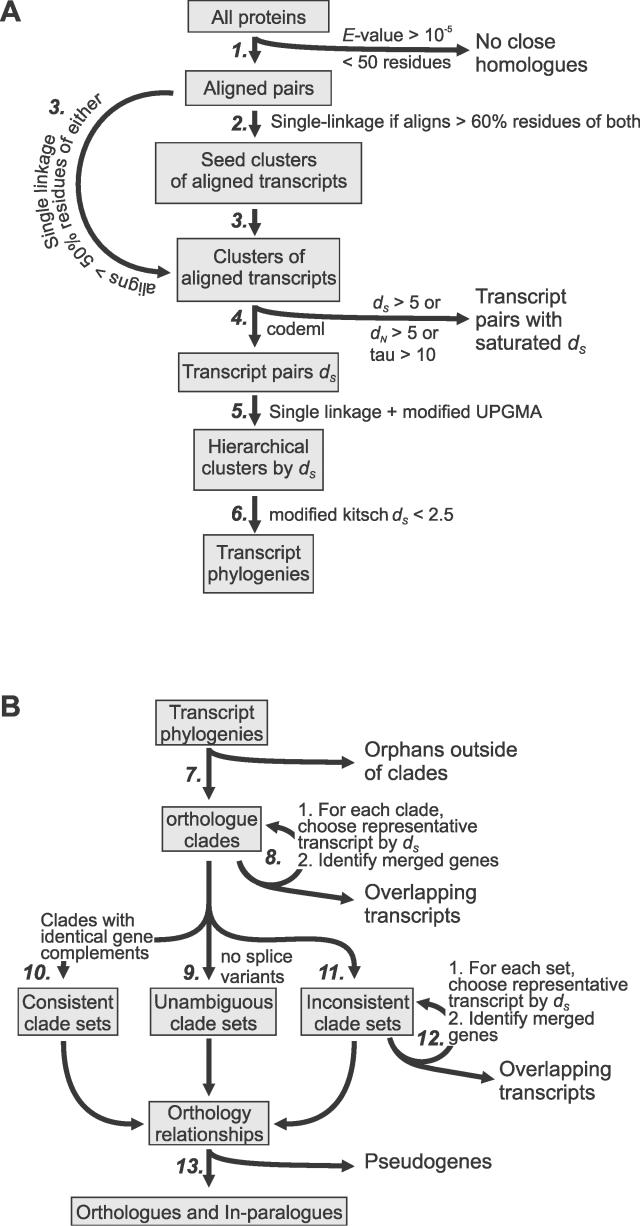
Overview of the PhyOP Orthology Prediction Process (A) Creation of transcript-based phylogenies. An all-versus-all BLASTP search is run for all proteins from two species (step 1) with an *E* value upper threshold of 10^−5^ and an alignment length threshold of 50 residues. Proteins pairs are linked together in initial clusters (step 2) if the alignment covers >60% of the residues of both sequences. Any remaining proteins are linked to the initial clusters if they align to >50% of the residues of either sequence (step 3). *d_S_* values are calculated from the pairwise alignments (step 4), and unsaturated transcript pairs (*d_S_* < 5.0) grouped first by single linkage and then hierarchically clustered using UPGMA (step 5). Phylogenies are created from cluster branches corresponding to *d_S_* < 2.5 by applying a modified version of the Fitch-Margoliash criterion (step 6). (B) Prediction of orthology from transcript phylogenies. Transcripts outside of clades of orthologous transcripts are discarded (step 7), and merged genes within orthologous clades are separated (step 8). Transcript clades were separated into three groups: unambiguous clades (step 9) containing genes with no other remaining splice variant; consistent sets of clades (step 10) with identical gene complements; and inconsistent clades (step 11) with different gene orthology relationships suggested by different sets of orthologous transcripts. The inconsistencies are resolved by separating merged genes and choosing transcripts with the lowest *d_S_* to its orthologous transcripts (step 12). Candidate pseudogenes are then discarded to give the final set of orthologous and paralogous genes (step 13).

Details of the derivation of transcript phylogeny by PhyOP are described in Materials and Methods and are illustrated as flow diagrams in Figure 2. Briefly, *d_S_* is calculated for all significantly high-scoring aligned transcript pairs from dog and human genomes. PhyOP constructs phylogenetic trees of transcripts by minimizing differences between the predicted branch lengths and pairwise *d_S_* using weighted least-square phylogenetic methods that ignore saturated *d_S_* values.

### Orthologue Predictions

Using the phylogeny of all transcripts, PhyOP was able to predict 14,807 dog genes in 1:1 orthologue relationships with human genes (Table 1). This involved 87% of all predicted dog genes. Together with dog orthologues in “many” relationships, this method predicts orthology for 93% of genes in the dog genome assembly. This is approximately twice the proportion previously predicted for rat and human genes [7]. These numbers exclude Ensembl gene predictions that are likely to be nonfunctional pseudogenes (see below).

**Table 1:**
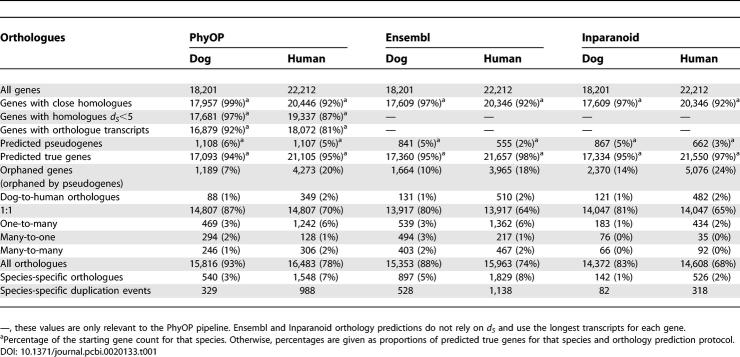
Numbers of Orthologues Predicted by PhyOP, Ensembl, and Inparanoid

### Orthologues from Consistent Phylogenies

In the vast majority of cases, even though orthologous genes may have multiple splice variants, only one transcript for each gene was found to be in an orthologous relationship. This allowed the orthologous relationships between genes to be inferred straightforwardly and with confidence. This was the case for 14,896 dog genes and 15,417 human genes. These include 465 dog and 1,286 human genes that were involved in lineage-specific duplications.

**Figure 3:**
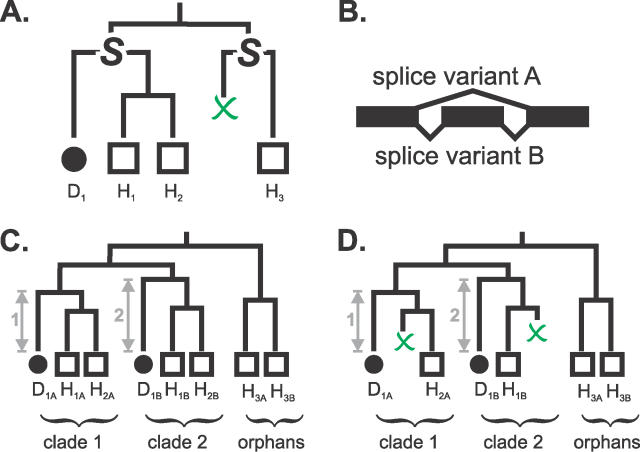
Deriving Orthology via Transcript Phylogeny (A,B) Phylogenetic relationships for a dog (D_1_) and three human (H_1_, H_2_, and H_3_) genes. D_1_ is the orthologue to H_1_ and H_2_. H_3_ has been orphaned by the loss of its dog orthologue. Each gene has two splice variants A and B (B), and their transcripts are subscripted accordingly. (C) Phylogenetic relationships for all transcripts. Each group or clade of orthologous transcripts recapitulates the gene orthology in (A). The transcripts A and B for the orphaned gene H_3_ are also themselves orphaned on the transcript tree. The transcripts from clade 1 are selected to represent the three genes (D_1_, H_1_, and H_2_) because phylogenetic distance between orthologues (arrow 1) is smaller than that for clade 2 (arrow 2). (D) How orthology is predicted when transcripts are missing. D_1A_ and H_2A_ are selected as the representative transcripts for their genes because the *d_S_* between these orthologues is smaller than that for D_1B_ and H_1B_. The transcripts in clade 1 are used to predict orthology between D_1_ and H_2_. Though H_1_ also has transcripts in orthologous relationships with D_1_, orthology between these two genes is not predicted, leaving H_1_ as an orphan. No orthology predictions are made for the gene H_3_, which remains as an orphan.

In only a surprisingly small number of cases (198 dog and 198 human genes) did genes possess multiple transcripts that were all in consistent orthologous relationships. These orthologues each have an identical number of transcripts, all in orthologous relationships (Figure 3). The rarity of such cases suggests either that it is difficult to correctly predict splice variants or that the exon architecture of a gene evolves rapidly, as has been reported by others [25,26]. For this small number of genes, we selected representative transcripts by applying a simple heuristic. We chose clades of transcripts with the smallest phylogenetic distance between orthologues (i.e., branch length from the root of the clade; Figure 3C), reasoning that transcript pairs with large calculated *d_S_* values are more likely to contain nonorthologous sequences.

### Orthologues from Inconsistent Phylogenies

720 dog and 859 human genes were predicted in orthology relationships following the resolution of inconsistent transcript phylogenies. These are cases where different combinations of orthology relationships between genes are suggested by different splice variants. In some instances, inconsistencies were due to missing transcripts (Figure 3D); in others, the transcripts may be truncated, or the underlying sequences may contain errors. It was important to resolve these difficult cases not only because they involved a significant number of orthologue gene candidates, but also because genes with lineage-specific duplications are disproportionately represented in this class. We resolved these phylogenetic inconsistencies by selecting, to represent each gene, a single transcript that has the shortest *d_S_* value to its orthologous transcript. The progressive elimination of transcripts inevitably meant that a few genes (40 and 139 from the dog and human genomes, respectively) with transcripts apparently in orthologous relationships nevertheless ended up as being “orphaned” (Figure 3D).

### Separating Merged Genes

**Figure 4:**
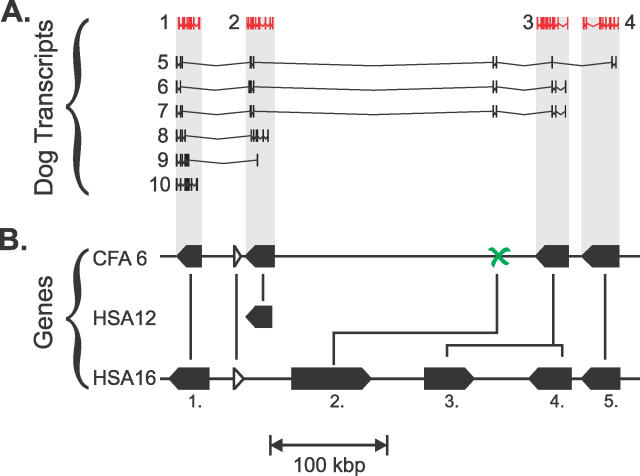
Distinct Dog Genes from Ensembl that Have Been Mispredicted as a Single Merged Chimera (A) Ten predicted transcripts for a single Ensembl dog gene (ENSCAFG00000017952) on CFA 6. PhyOP orthology predictions suggest that only transcripts 1–4 highlighted in red are correct, and that these represent four distinct nonoverlapping dog in-paralogues (shaded in grey). Resolution of the transcript phylogeny strongly indicates that this one predicted gene is instead a composite of four true paralogous genes (in red; ENSCAFT00000028541, ENSCAFT00000028547, ENSCAFT00000028555, and ENSCAFT00000028561) and one pseudogene. At least five of the transcripts are chimeric constructs of exons from separate genes. In each and every case we examined, putative merged genes were the result of chimeric predicted transcripts sampling different combinations of exons from adjacent true paralogues. (B) The corresponding genomic region on CFA 6 with the distinct genes and their transcriptional orientations indicated by the black pentagons. Below this is the orthologous genomic region from HSA 16 showing five human orthologues (numbered 1–5: ENSG00000005187, ENSG000000166743, ENSG000000166747, ENSG000000066813, and ENSG000000183549). The orthology predictions are indicated with solid black lines. Thus, the dog orthologue for transcript 3 (gene 4) has acquired an extra tandem duplicate (gene 3). Only fragmentary exons on dog CFA6, corresponding to a pseudogene (marked with a cross), can be found for human gene 2, which, therefore, is assigned as an orphan. The human orthologue for the dog gene for transcript 2 unusually appears to have been translocated to HSA 12, as corroborated by BLASTZ [64] genome alignments. Apart from this, gene order and strand have been conserved among orthologues of both lineages, including those for an unrelated orthologue pair (hollow triangles) in the middle of the paralogue cluster (ENSCAFG00000017985 and ENSG000000066654).

We found 388 dog and 322 human gene predictions that appear to have been erroneously merged with neighbouring paralogous genes, although a small minority of these might represent chimeric gene fusions [27–29]. Such instances were evident among genes whose transcripts were placed in inconsistent phylogenies (Figure 4).

We disentangled merged genes systematically as part of the orthology prediction method. Proper resolution of the transcript phylogeny exploited the observation that transcripts derived from merged genes are chimeric: they possess both orthologous and paralogous regions with respect to transcripts from the other species, and thus tend to exhibit elevated *d_S_* values and hence long branches. Most such problematic transcripts are, in fact, automatically rejected as “orphans” (i.e., those not in orthologous relationships with any other transcript) by our procedure.

Using the previously described criteria, we selected a representative transcript while simultaneously discarding all other transcripts from the same gene with which it overlaps on the genome (see Figure 4B). Remaining transcripts are then treated as candidates for a newly separated gene. The representative transcript for this new gene can be chosen in turn (using the smallest *d_S_* to remaining orthologues in the same clade), and further candidate transcripts representing more merged genes identified, if necessary. The separation of erroneously merged predictions resulted in the prediction of 429 and 584 additional dog and human orthologue genes that otherwise would have remained as orphans.

### Pseudogenes

An initial survey of predicted in-paralogues indicated significant contamination with processed pseudogenes. These are widely dispersed, intron-less, or disrupted copies of known multi-exonic genes, and are due to the retrotransposition of mature RNAs. Since there are an estimated 19,000 pseudogenes in the human genome [30], it is unsurprising that some of these should appear among the predicted gene set. Homologues of highly expressed ribosomal and RNA- and DNA-binding proteins are especially numerous among paralogous retrogenes. These have previously been shown to be overrepresented among pseudogenes [30–32], presumably because of the high expression of such genes in germline cells. As a result, predicted in-paralogues are more likely to have reading frame disruptions and single exons, and be located far from conserved syntenic regions (Table 2).

**Table 2:**
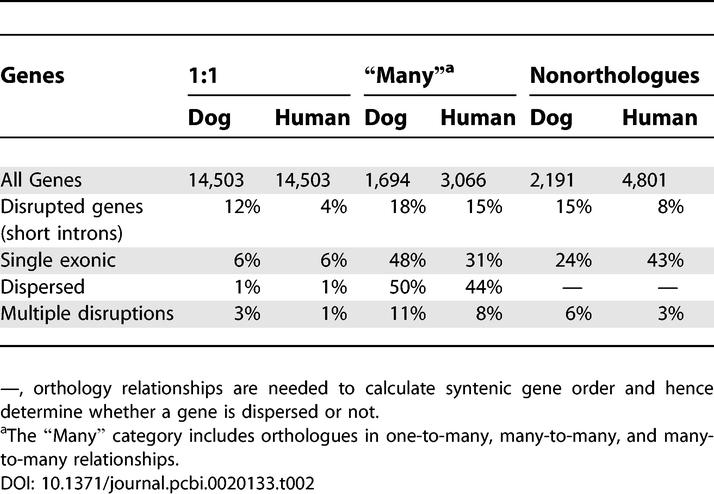
Evidence from PhyOP for Pseudogenes among Ensembl Gene Predictions

We used the following heuristic to filter out these retrogenes. All single-exonic or disrupted genes found outside syntenic blocks were discarded. Genes with multiple disruptions were also discarded as nonfunctional. In addition, for large orthologue sets with widely scattered members (on more than four chromosomes), we identified the orthologues (at least one from each species) most likely to represent true genes (using the criterion of three or more exons with matching exon boundaries in both species) and excluded all other orthologues with few (less than three) and nonmatching exons.

**Figure 5:**
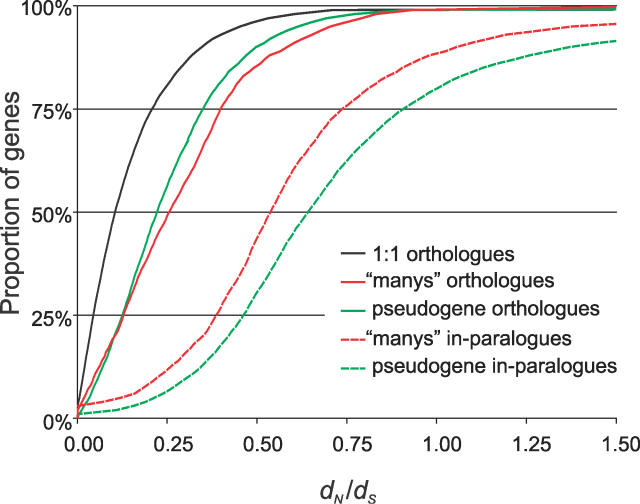
*d_N_/d_S_* Cumulative Frequency Distribution for Orthologues, Paralogues, and Pseudogenes Predicted by PhyOP Predicted pseudogenes exhibit median *d_N_/d_S_* ratios of 0.22 when compared with their orthologues, 0.55 with functional in-paralogues, and 0.65 with in-paralogues that are themselves also candidate pseudogenes. The 1:1 orthologues have a median *d_N_/d_S_* of 0.11. Assuming a constant mutation rate, the *d_N_/d_S_* after loss of function in pseudogenes should relax towards approximately 0.55 (the average of 1.00 for no selection and 0.11 for purifying selection) when compared with a functional homologue, and towards 1.00 when compared with a homologue which is also a pseudogene. The *d_N_/d_S_* distribution between in-paralogues (dashed lines) is greatly shifted upwards, suggesting that the changes in selective constraints for both functional and pseudogene paralogues tend to be much more recent than the dog–human divergence.

Altogether, we used these criteria to identify 1,108 dog and 1,107 human candidate pseudogenes (Table 1) that, as expected, show considerable relaxation of selective constraint (Figure 5). Their gene ontology (GO) annotations are significantly overrepresented (*p* < 0.05) in the terms for “ribosome,” “RNA binding,” and “protein biosynthesis” (Table 3).

**Table 3:**
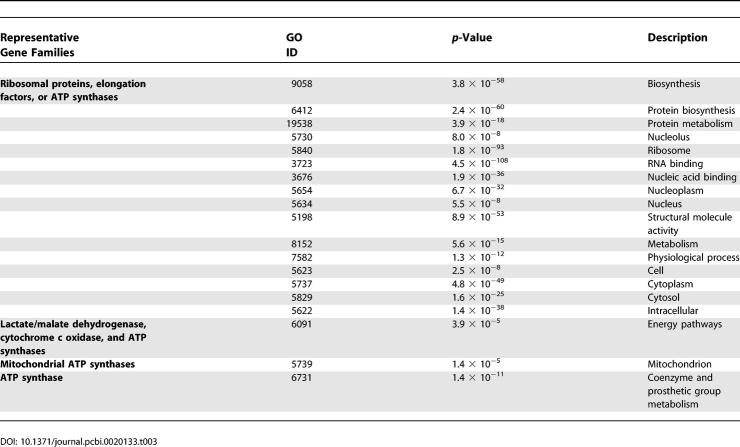
Overrepresented GO Categories among Putative Pseudogenes

Removal of these putative pseudogenes also left orphaned 88 dog and 349 human genes. Our lists of pseudogenes necessarily include functional genes that have multiple apparent disruptions due to sequencing or gene prediction errors. Since these are far more common in the incomplete dog genome assembly, there should be more true dog genes erroneously predicted as pseudogenes, and also more human than dog orthologues orphaned by pseudogenes.

### Quality of Orthologues

Several independent measures show that orthology predictions produced by PhyOP are of high quality. Protein sequences corresponding to the representative transcripts of these 1:1 orthologues are aligned essentially throughout their entire lengths, and 92% have well-conserved exon boundaries (see Materials and Methods). Careful manual examination of selected genes suggests that most of the remaining discrepancies either derive from our conservative approach in comparing exon structure (some real changes in exon lengths have occurred since the human–dog divergence) or are due to errors in the prediction of gene structure, such as missing exons or extra introns. It should be noted that gene predictions for the dog genome are more challenging given both the paucity of dog mRNA data and the draft quality of the canine genome assembly.

### In-Paralogues

The PhyOP pipeline predicted 540 dog and 1,548 human in-paralogues, representing 329 dog and 988 human duplication events (Table 1). Human gene duplications appear to have been fixed at a rate three times higher than in the dog lineage (see Discussion).

**Figure 6:**
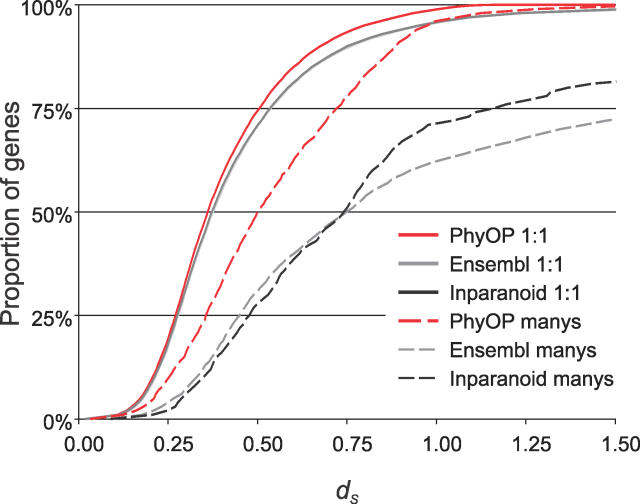
PhyOP, Ensembl, and Inparanoid *d_S_* Cumulative Frequency Distributions These include orthologues which have (manys) or have not (1:1) been involved in lineage specific duplications. The *d_S_* distributions for 1:1 orthologues are similar for the three methods. The distributions for Ensembl and Inparanoid 1:1 orthologues are indistinguishable, and the median *d_S_* for PhyOP 1:1 orthologues is only slightly smaller. This is mainly because most of the predictions are common to all. PhyOP “manys” orthologues have a larger median *d_S_* than do 1:1 orthologues. The *d_S_* distributions for “manys” orthologues predicted by Inparanoid and Ensembl are very much shifted to the right, indicating that a large proportion of these genes may have diverged well before the dog and human lineages separated.

In-paralogues have significantly lower percentages of identity (median of 78.1% versus 91.8% in 1:1 orthologues) and higher *d_N_*/*d_S_* ratios (median of 0.23; Table 4 and Figure 5), where *d_N_* is the number of nonsynonymous substitutions per nonsynonymous substitution site. These suggest relaxation of evolutionary constraints or adaptation after gene duplication [33–35]. Orthologues with lineage-specific duplications also tend to have larger *d_S_* values than 1:1 orthologues (Table 5 and Figure 6). This may be because the frequency of segmental duplications (which often generate gene paralogues) is positively correlated with *d_S_* [36]. It has also been suggested that an increased *d_N_*, such as that seen in duplicated orthologues, can lead to higher *d_S_* via mutational influences of 5′- and 3′-flanking bases [37,38].

**Table 4:**
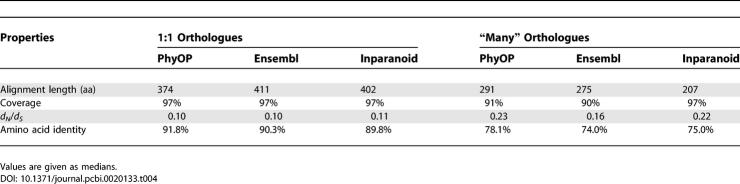
Properties of Orthologues Predicted by PhyOP, Ensembl, and Inparanoid

**Table 5:**
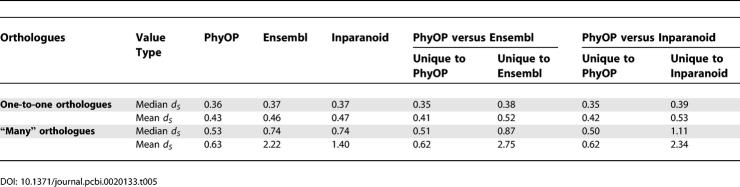
Median and Mean *d_S_* Values of Orthologues Predicted by PhyOP, Ensembl, and Inparanoid

### In-Paralogues Tend To Be Shorter than 1:1 Orthologues

Most duplicated genes exhibit full-length alignments with their orthologues (median, 91%). Transcripts of in-paralogues, however, tended to be considerably shorter than those in 1:1 relationships, to encode shorter peptides, and to comprise fewer exons (medians of two and four for dog and human in-paralogues, compared with medians of seven and eight for dog and human 1:1 orthologues, respectively; Table 6). In-paralogues were also more likely to be single-exonic, which appears from close inspection of individual cases (including, for example, olfactory receptor and α-interferon genes) not to be due to contamination with large numbers of likely pseudogenes. Compared with 1:1 orthologues, in-paralogues are considerably less likely to possess conserved exon boundaries (67% versus 92% in 1:1 orthologues), perhaps reflecting the greater challenge in predicting adjacent sequence-similar paralogues.

**Table 6:**
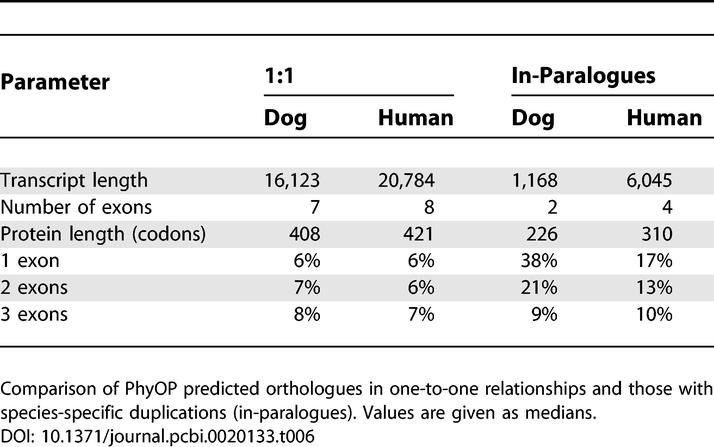
In-Paralogues Are Smaller than One-to-One Orthologues

The evidence thus suggests that there is a mutational bias towards shorter in-paralogues. Duplicated genomic segments in many species tend to have an L-shaped distribution curve biased towards short regions [39,40], and a majority of segmental duplications in the human-lineage are smaller than the median gene size ([40] and Table 6). Longer genes may be less likely to be duplicated in their entirety with promoter and multiple exons intact and may be more likely, instead, to give rise to nonfunctional gene fragments.

### Orphaned Genes without Predicted Orthologues

A minority of genes (1,189 dog and 4,273 human genes) did not possess any transcripts in orthologous relationships and were classified by PhyOP as “orphans.” These are genes in which the corresponding copy has either been lost or has failed to be predicted in the other lineage.

Many of these orphans may not represent functional protein-coding genes. Instead, they include chimeric transcripts or even non–protein-coding sequences as a result of assembly or gene prediction errors. cDNAs generated by high-throughput projects are occasionally incomplete, consisting only of the untranslated regions. This leads to spurious open reading frames being called within the untranslated region and submitted to protein databases as genuine coding transcripts (Ewan Birney, personal communication). These various types of defective genes would all tend to have increased *d_S_,* suggesting an ancient divergence from any other partially homologous sequence. This would be consistent with the large proportion of orphan genes that are single-exonic (24% and 43% out of dog and human orphans, respectively, versus 6% of 1:1 orthologues) and the overrepresentation of genes with multiple apparent frame disruptions in the dog genome (6% of orphans versus 3% of 1:1 orthologues). Other orphans, especially in the dog genome, appear to represent genes that have been predicted only as multiple fragments (e.g., the dog gene fragments corresponding to the human titin *[TTN]* gene).

Few large genomic regions in either species were without orthologues, confirming the general high quality both of the dog assembly (CanFam 1) used for the gene build, and of Ensembl's predicted gene set. There were only two regions in the human genome that contained 20 or more orphaned genes in the dog (containing 22 and 24 genes, respectively). The largest number of consecutive dog genes without a predicted human orthologue was only 11. (This is despite the many human genes [1,766 ] without close dog homologues, using a BLAST upper threshold of <10^−5^.)

### Estimating the Human Gene Count

Our procedure for distinguishing pseudogenes and our discovery of apparently chimeric merged gene predictions necessitates a readjustment of the number of functional protein coding genes as identified by the Ensembl gene prediction pipeline. With a starting human gene set of 22,212, adding 164 previously merged genes and removing 1,107 pseudogenes gives a revised gene count of 21,269. However, many of the orphans are likely also to be nonfunctional, as discussed above. The number of fragmentary and nonfunctional genes among orphans can be estimated simply from the excess of single-exon orphaned gene predictions compared with 1:1 orthologues (Table 2): (42.6% − 6.4% = 36.2%) of 4,273 orphans ≈ 1,550 pseudogenes. This provides an estimate of 19,700 functional human protein coding genes predicted by Ensembl.

This rough estimate assumes that all the putative retrotransposed genes we have identified are nonfunctional and that, conversely, most of the orthologues we predict represent real genes and not pseudogenes. Nevertheless, this number is similar to the predicted protein-coding gene count of nematode worms [14] and a protein-coding human gene count estimated using an independent comparative approach (19,400: Michele Clamp, personal communication).

### Orthologous Chromosomal Segments

**Figure 7:**
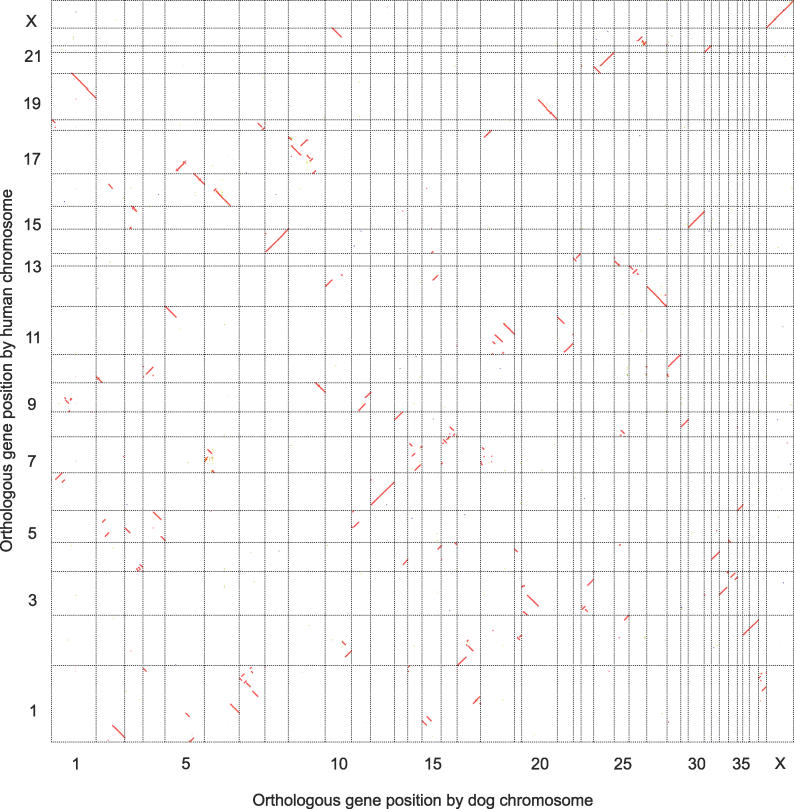
Oxford Grid of PhyOP Orthologues Showing Dog–Human Genomic Synteny Genes are plotted in consecutive gene order along the dog chromosomes CFA 1–38 and CFA X, and along the human chromosomes HSA 1–22 and HSA X. One-to-one, one-to-many, many-to-one, and many-to-many dog-to-human orthologues are displayed as red, green, blue, and black dots, respectively. Diagonal lines represent genomic segments with conserved synteny.

The high coverage and accuracy of PhyOP allowed us to create a gene-based map of conserved synteny between dog and human genome assemblies (Figure 7). Previous attempts at deriving gene-based synteny maps [41] have relied on reciprocal best hits, and so are expected to exhibit many problems at high resolution (see Benchmarking below).

By analogy with whole-genome alignment methods [6], we defined a micro-syntenic segment to be a chromosomal region from one species that contains genes whose orthologues occur in the same order and transcriptional orientation on a single chromosome of the other species. A macro-synteny block comprises one or more micro-syntenic segments that are contiguous in both species but which might be rearranged in order or in orientation.

Mapping the dog and human PhyOP orthologues to their genome assemblies revealed 178 dog and 192 human macro-synteny blocks. Half of all orthologues reside in macro-syntenic blocks of 145 and 167 genes or larger in the dog and human genomes, respectively. Gene order is, in the main, highly conserved across the dog and human genomes since few genes reside in small blocks: <1.2% can be found in small blocks containing fewer than ten orthologues. In particular, dog chromosomes CFA29, CFA30, CFA32, CFA36, and CFAX are all completely syntenic to regions of HSA8, HSA4, HSA6, HSA2, and HSAX, respectively; and CFA12, CFA21, CFA24, CFA28, CFA33, CFA35, and CFA38 are orthologous to regions of HSA6, HSA11, HSA20, HSA10, HSA3, HSA6, and HSA1, respectively (Figure 7). Another 14 dog chromosomes possess macro-synteny blocks orthologous to only two human chromosomes each. These findings recapitulate canine synteny maps derived from reciprocal chromosome painting [42], radiation hybrid mapping [43], and unique sequence alignments [24].

**Figure 8:**
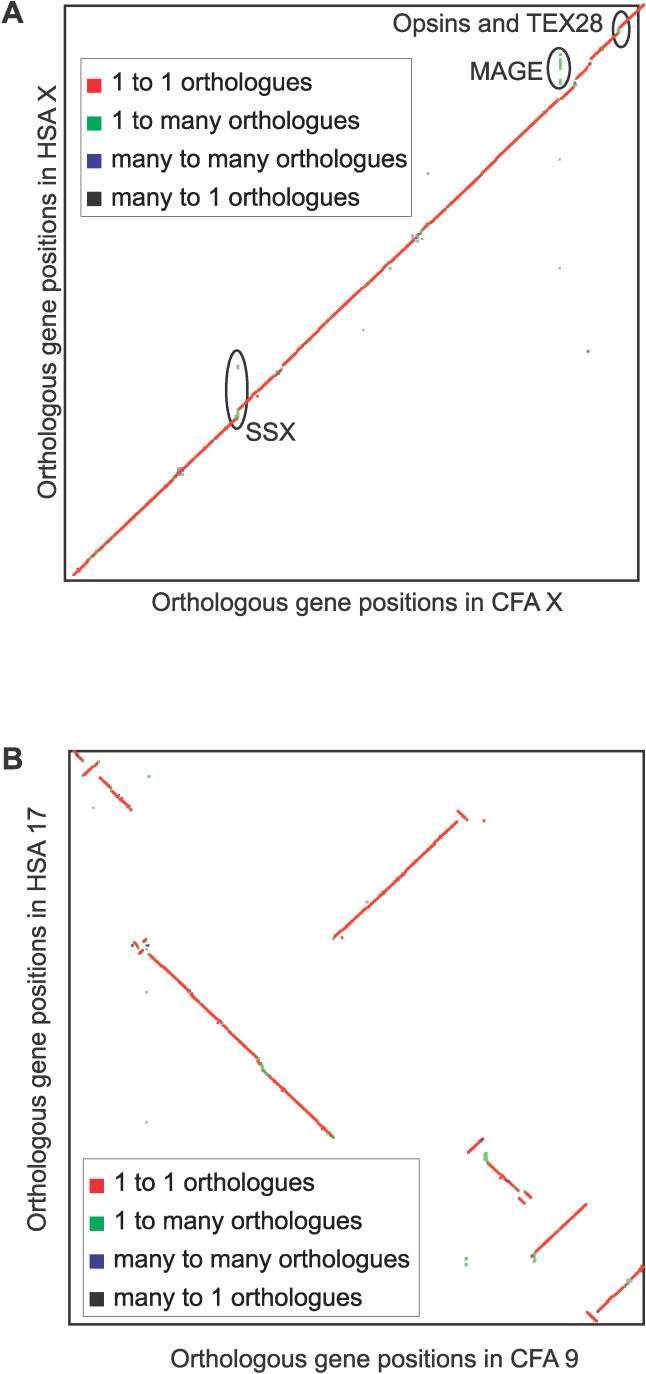
Dotplot of PhyOP Orthologues Showing Conserved Synteny in the Dog and Human Chromosomes (A) Synteny between CFAX and HSAX. (B) Synteny between CFA9 and HSA17. Genes are plotted in consecutive gene order along each chromosome. The two X chromosomes are in a single conserved syntenic block. However, known human-specific paralogues of SSX, MAGE, opsin, and TEX28 families have been highlighted. The sequence containing the opsin and TEX28 families is highly polymorphic in the human population [65]. The human X chromosome genome sequence contains two copies of the green-cone photoreceptor pigment gene in the opsin family interdigitated with three full-length copies of TEX 28. The plot of orthologous gene positions between CFA9 and HSA17 recapitulates known syntenic rearrangements in the human lineage [6].

Collinear gene order is conserved at larger distances, including over entire lengths of the X chromosomes (Figure 8A), as expected [44,45]. Nevertheless, within each macro-syntenic block, typically there has been much rearrangement in both order and orientation, with parsimony suggesting multiple chromosomal inversions in either dog or human with respect to the ancestral chromosome (Figure 8B). Thus, half of PhyOP orthologues (N_Orth,50_) reside in stretches of only 48 genes or more that retain gene order and orientation.

In-paralogues are much more likely to be found in smaller micro-syntenic blocks, probably because both gene duplications and chromosomal rearrangements are correlated with the rate of chromosome breakage [46]. The corresponding N_Orth,50_ values for such dog and human genes are only 25 and 15 (Table 7).

**Table 7:**
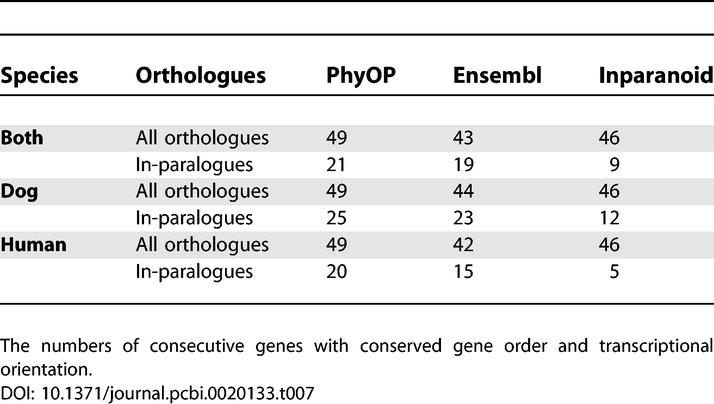
Median Micro-Syntenic Block Sizes for PhyOP, Ensembl, and Inparanoid

### Benchmarking PhyOP with Ensembl and Inparanoid Methods

We compared the *d_S_*-based orthologue predictions by PhyOP to two other sets predicted on the basis of protein similarity: the first set from Ensembl's Compara database [11], and the second predicted using Inparanoid [2]. Initial orthologue sets for both Ensembl and Inparanoid are founded on protein sequences which are the reciprocal BLASTP [12] best matches of each other. These are described by Ensembl as UBRH or MBRH, for unique or multiple best reciprocal hits [13]. Ensembl also incorporates additional nonreciprocal best matches (RHS, or reciprocal hit based on synteny information, in Ensembl nomenclature) if both genes are less than 1.5 Mb away from a pair of BLAST reciprocal best matches. As described in the Introduction, RHS genes are not derived from a reciprocal procedure and many exhibit nontransitive and conflicting relationships (Figure 1). We resolved such contradictions in the phylogenetic relationships by grouping Ensembl orthologues using single linkage. We used the same criteria described above for PhyOP to exclude putative pseudogenes from among the Ensembl and Inparanoid orthologues.

**Figure 9:**
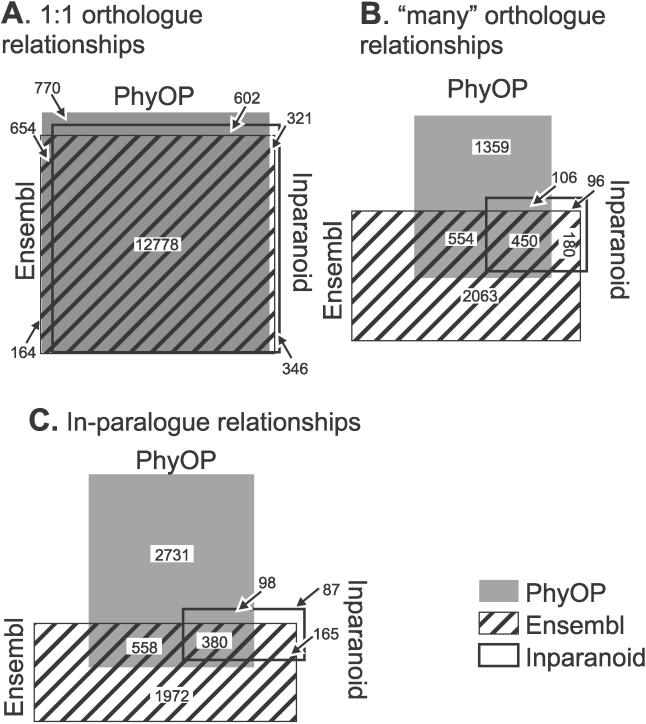
Venn Diagram Comparing Orthology Relationships Predicted by PhyOP, Ensembl, and Inparanoid (A) Most 1:1 orthologue predictions are shared between the three methods: PhyOP (solid rectangle), Ensembl (striped rectangle), and Inparanoid (hollow rectangle). (B) Orthology predictions that involve lineage-specific duplications, however, differ markedly between PhyOP and Ensembl. Most Inparanoid predictions are a subset of those from Ensembl. (C) The same is true for predicted paralogy relationships.

The three methods, PhyOP, Ensembl, and Inparanoid, predicted similar numbers of 1:1 orthologues (14,807, 13,917, and 14,047). The three sets of predictions largely overlapped, with 12,778 common to all three methods (Figure 9A), resulting in similar median *d_S_* among the three methods (Table 5). However, 1:1 orthology relationships that are predicted only by Ensembl or Inparanoid are more diverged than expected, with 25% higher mean *d_S_* values (Table 5). Conversely, the *d_S_* values for the additional 2,029 orthology relationships predicted only by PhyOP are indistinguishable from those of orthologues predicted by all methods. This indicates that 1:1 relationships unique to PhyOP are more reliable than those of the other two methods.

### PhyOP In-Paralogues Are Very Different from Ensembl and Inparanoid Predictions

However, orthology predictions where duplications have occurred in the dog or human lineages (i.e., those in one-to-many, many-to-one, or many-to-many relationships) differ significantly among the three methods. PhyOP predicts 2,469 such relationships, compared with 3,247 for Ensembl and only 832 for Inparanoid. The majority (88%) of Inparanoid orthologues in “many” relationships are a subset of those from Ensembl, but PhyOP predictions are strikingly different from either (Figure 9B). This is also the case for in-paralogue relationships predicted by the three processes (Figure 9C**)**. Inparanoid predictions are largely a subset of Ensembl predictions (75%), while the majority (46%) of PhyOP paralogy relationships are unique to this method.

The orthologues in “many” relationships predicted by the three methods had similar values for protein sequence coverage and percentage identity (Table 4), but Inparanoid alignments were noticeably shorter (median lengths of 207 residues versus medians of 291 and 275 for PhyOP and Ensembl). The *d_S_* distribution curves for Ensembl and Inparanoid “many” orthologues were greatly shifted to higher values (dashed lines in Figure 6), each with a median *d_S_* value of 0.74, and they include significant proportions (19% for Ensembl and 14% for Inparanoid orthologues) with saturating *d_S_* values >> 2.5. By comparison, the median *d_S_* value for PhyOP “many” orthologues was 0.53. The higher *d_S_* for many of the Ensembl and Inparanoid predicted orthologues explains why PhyOP did not consider these relationships (Table 5). In particular, both Inparanoid and Ensembl include predicted human- and dog-specific duplications that, despite sharing 100% percentage protein sequence identity, appear, from their large number of synonymous substitutions, to have been present in the common ancestor of the dog and human. Such instances include genes encoding histones and the calmodulin (delta) subunit of phosphorylase *b* kinase. In these cases, Ensembl and Inparanoid appear to have been misled into predicting recent, rather than ancient, divergence by strongly purifying pressure that has conserved protein sequence.

### Conservation of Gene Order among PhyOP, Ensembl, and Inparanoid Predictions

If most genes are duplicated in local tandem copies, and if the rate of genomic rearrangement is low relative to that for gene duplication, then most orthologues would tend to be conserved in gene order. Consequently, we sought to use conserved synteny as a useful benchmark for determining the reliability of each method. We used the size of the micro-synteny segments (those with conserved gene order and transcriptional orientation) as a measure of conservation of ancestral gene order. We found that PhyOP orthologues are more likely to have conserved gene order between dog and human (N_Orth,50_ of 48), with Inparanoid (N_Orth,50_ of 46) and Ensembl (N_Orth,50_ of 43) orthologues more likely to be found in smaller segments (Table 7). We also wondered whether the “many” orthologues common to Ensembl, Inparanoid, and PhyOP would be more reliable than those of any one alone. We found, however, that PhyOP orthologue predictions that were also shared by the other two approaches had exactly the same N_Orth,50_ of 48.

### Lineage-Specific Biology

Though duplications appear to have been fixed at a higher rate in the human lineage than in the dog, the functional classes of genes involved are very similar. In both species, genes involved in immunity, chemosensation, and reproduction are prominent (Tables 8 and 9), much as has previously been observed for other mammalian species [3,6,7]. A significant number of dog and human in-paralogues appear to involve independent duplications in both the genomes (46% and 20% of dog and human in-paralogues). It is likely that some of these cases represent gene conversions of more anciently diverged out-paralogues in both species, while others represent true independent duplications arising from common selective pressures on both lineages.

**Table 8:**
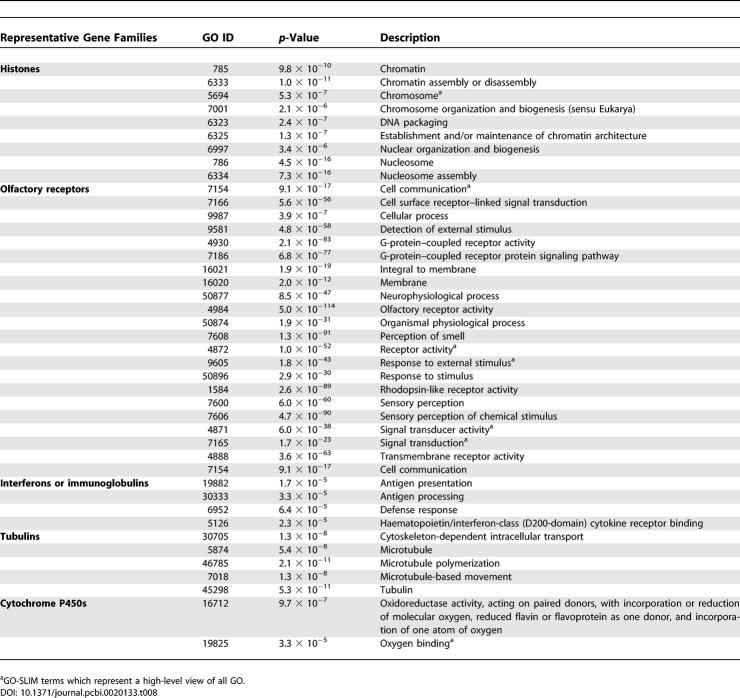
Overrepresentation of GO Categories among Dog In-Paralogues

**Table 9:**
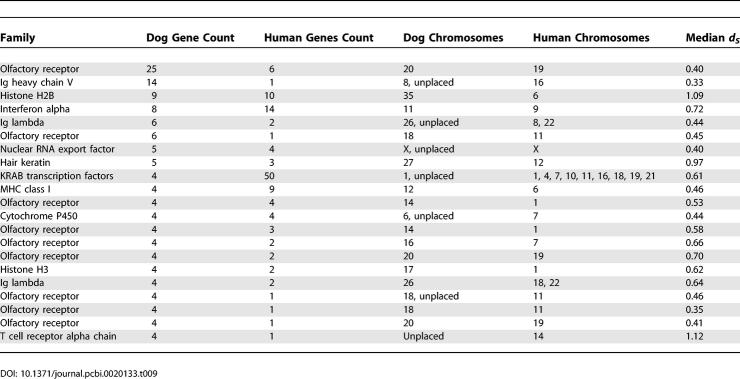
Large Families of Dog In-Paralogues

In the main, gene duplications have generated in-paralogues that lie in tandem in the extant genomes. The striking exception to the close physical proximity of in-paralogues is the human-lineage-specific duplication of KRAB-zinc finger (KRAB-ZnF) genes [47]. The ancestral genes, which have been inherited without dispersal in the dog lineage on CFA1, have been duplicated onto twelve human chromosomes (Table 9). Dispersal in the human lineage has not involved retrotransposition as KRAB-ZnF gene structures have been preserved. What then might have caused the unusual dispersal of these genes? One possibility is that these genes lie in sequence that has been especially susceptible to duplication. However, it is also possible that the disruption of physical linkage between in-paralogues might have proved advantageous. This might be because selection on closely linked genes is often less efficient (the Hill-Robertson effect [48]): KRAB-ZnF genes often appear to be under positive selection [6,47]. However, the functions of primate KRAB-ZnF genes remain obscure and the molecular and cellular basis for their proposed adaptive events remain to be determined.

## Discussion

We have presented a new phylogenetic method, PhyOP, which has succeeded in predicting human orthologues for 93% of dog genes. The 1:1 orthologues predicted by PhyOP appear to be more comprehensive and more accurate than those of Inparanoid and Ensembl. The method's major advances, however, are in the predictions of in-paralogues and transcript phylogenies. In-paralogues predicted by PhyOP are more numerous, are less divergent at synonymous sites, and better recapitulate conserved synteny than either Ensembl or Inparanoid. Consistent orthology, including the conservation of intron–exon boundaries, may be useful in detecting mispredicted and nonfunctional genes, and we have identified numerous chimeras and candidates for pseudogenes in the dog and human genomes.

### Human Gene Duplications Are More Numerous

There is a considerable disparity between the numbers of dog and human in-paralogues. It appears that the human lineage has accumulated 3-fold more gene duplicates than has the dog lineage. This may be a result of the lower rates of repeat-mediated segmental duplication in the dog lineage [49] associated with the almost 10-fold lower activity of endogenous retroviral and DNA transposons compared with that in the human [24]. It is also likely that some duplicated genomic regions have been collapsed in the draft assembly of the dog genome.

There is, however, an alternative explanation for the larger number of gene duplicates in the human genome assembly: many gene duplicates represented in the human genome assembly may not have been fixed in the population. Rather, they are copy number variants whose appearance in the human genome assembly reflects the mosaicism of the human reference sequence, with contributions from the diverse haplotypes of each of the multiple sequenced individuals. Certainly the majority of duplicates are of recent provenance because their divergences are extremely low [23]. By contrast, the dog genome has been assembled from only a single inbred boxer dog without the incorporation of copy number variants from other dogs.

### Characteristics of In-Paralogues

Most in-paralogues in the dog and human lineages are found in tandem arrays, though human in-paralogues are more likely to have dispersed either to beyond 20 genes from their conserved syntenic gene position on the same chromosome (5%) or else to another chromosome (9%). Again, this may be related to higher rates of repeat-mediated human segmental duplication.

In both species, in-paralogues appear to be enriched in genes with few exons. There are overrepresentations of short genes, including single-exonic genes, and those with two or three exons, which are positioned in conserved synteny and thus are unlikely to be nonfunctional retrogenes. The preponderance of short genes would be explained by the relative infrequency of segmental duplications that are sufficiently large to completely encompass sprawling multi-exonic genes, including their 5′ and 3′ regulatory regions [40].

In-paralogues also appear to exhibit higher apparent mutation rates. Estimated *d_S_* values between orthologous genes are higher if these have contributed to lineage-specific duplications (Figure 6). There may be several reasons for this. First, in-paralogues tend to evolve faster, either because of relaxed purification selection or adaptation [34]. Because of mutational dependences of adjacent residues, especially at sites involving methylated CpGs, an elevated nonsynonymous rate can also result in higher *d_S_* values [37,38]. Second, biased gene conversion, especially between in-paralogues in tandem copies, can increase the number of synonymous substitutions [50,51]. The resulting increased G + C content may also bias the calculated *d_S_* towards higher values, notwithstanding that the maximum likelihood estimation of *d_S_* takes codon usage into account. Finally, it is possible that in-paralogues are underrepresented in housekeeping genes that are expressed in the germline. In-paralogues may therefore be less likely to be subject to transcription-coupled repair processes [52,53] that act to reduce the mutation rate.

The genes that have duplicated in the dog lineage often possess functions in immunity (e.g., α-interferons), chemosensation (e.g., olfactory receptors), and toxin degradation (e.g., cytochrome P450s), categories which are enriched among gene duplications in other mammalian lineages. Nevertheless, the infrequency of dog gene duplications in an evolutionary lineage that has experienced great variation in anatomical morphology indicates that much developmental change may arise not by gene duplication but within the non–protein-coding regulatory segments of the mammalian genome.

### Alternative Distance Metrics

Although, we have used *d_S_* values as a proxy for neutral rates in the analysis of the dog and human genomes, PhyOP can also make use of other similar measures. These include divergence of ancestral repeats or of the interiors of introns, which are relatively free of functional constraints [54,55]. We have also shown separately that PhyOP can accurately infer relationships between more divergent genes and species using amino acid–based distances (unpublished data).

### Conclusion

The PhyOP pipeline has provided robust and high-quality orthology and paralogy predictions for the dog and human genomes. However, this approach is also eminently suitable for unravelling the relationships between genes from multiple species simultaneously. Pairwise orthology prediction inaccuracies are additive, and the performance deteriorates with each additional species. Phylogenetic predictions, in contrast, grow more reliable as additional data from each genome allow previous gaps, due, for example, to gene deletions, to be illuminated. Because PhyOP does not use synteny information to predict orthology, it would also be applicable to partially assembled, incompletely sequenced genomes. In the case of the mammalian genomes currently being sequenced at low (~2-fold) statistical coverage [15], only ~86% of the bases in each genome will be covered, leading to many missed genes from each of the sequenced species. This will greatly complicate pairwise assignment of orthology by Ensembl or Inparanoid. PhyOP, using a fully phylogenetic approach to analyse the cohort of genomes simultaneously, should be highly reliable even in the face of missing genes.

## Materials and Methods

### Conventions

In this article, we indicate the class of orthology relationship by counts of dog orthologues followed by human unless otherwise specified. Thus, a one-to-many relationship refers to a single dog gene that is orthologous to several human-specific duplications. “Genes” in this article always refers to protein-coding genes.

### Identification of homologues

We collated all human and dog peptide sequence predictions from Ensembl (EnsMart version 27.1). Homologues were identified and aligned using BLASTP [12] using an *E* value upper threshold of 1 × 10^−5^. Alignments with fewer than 50 aligned residues were discarded. BLAST results are occasionally asymmetric due to heuristic failure. In such cases, we therefore always used the alignment with the higher bit score.

### Deriving gene phylogenies via transcript phylogenies

Our method assigns phylogenetic relationships among all transcripts for two species. From this transcript phylogeny, we reconstructed a second phylogeny, that for genes, to predict their orthology. Partial alignments (such as those between shared domains) can result in sprawling, transitively linked clusters of up to 10,000 transcript sequences. To overcome this, we seeded transcript clusters by single linkage, joining pairwise relationships where the alignments cover at least 60% of the residues in both sequences (Figure 2A, step 2). To avoid discarding fragmentary gene predictions, we then added unclustered transcripts to any seed cluster if they aligned to a cluster member over more than 50% of the residues of either sequence (Figure 2A, step 3). Further clusters were created from previously unconnected transcripts by single linkage clustering using the same 50% threshold. As a result, some transcripts are members of multiple clusters. Their true orthology remained to be disambiguated in subsequent steps using *d_S_* values. Corresponding protein-coding DNA sequences were retrieved from Ensembl and were aligned according to the amino acid pairwise alignment. *d_N_* and *d_S_* were calculated for the aligned regions using the codeml programme from the PAML package [56], with default settings for pairwise analyses and nine free parameters used to account for codon frequencies (F3X4; [57]).

### *d_S_*-Based phylogenies

Our simulations using the Evolver programme from the PAML package [56], and codon frequencies derived from the dog and human genomes, show that codeml is able to reliably estimate *d_S_* values up to 2.5 (unpublished data). For *d_S_* values between 2.5 and roughly 5.0–6.5, codeml is still able to give informative estimates (mean and median values are within 5%), but these are prone to increasingly larger errors due to saturation at synonymous sites. Accordingly, we disregarded all sequence comparisons which resulted in *d_S_* > 5.0. In addition, we biased our calculations so that larger *d_S_* values more prone to errors were down-weighted.

Phylogenies were built from sets of sequences related by reliable *d_S_*. These were obtained by clustering sequence pairs first by single linkage and then using a modified version of the UPGMA algorithm (Figure 2B, step 5). This latter method was adapted to ignore missing values. Each set of sequences represents branches of the UPGMA tree with a root-to-leaf branch length of less than 1.25 and thus corresponds to *d_S_* values of < 2.5.

*d_S_*-based distance matrices for these sequence sets frequently contain missing values. These can occur when sequences represent gene fragments or alternative transcripts so that some sequence pairs either do not overlap or result in alignments that are too short. However, the majority of missing values in the distance matrix are due to the discarded large *d_S_* values (>5.0) representing long branches.

Most popular distance-based methods, for example, neighbour-joining and BioNJ, require complete matrices without any missing values. The simulations of Makarenkov and Lapointe [58] show that weighted least-squares algorithms such as the Kitsch or Fitch programmes from the PHYLIP suite of programs [8] are clearly the most effective way to recover underlying phylogenies for incomplete matrices with missing values. Such approaches make it easy to accommodate the rapidly increasing errors as *d_S_* approaches saturation at large values.

### Modifying the Fitch-Margoliash criterion for incomplete distance matrices (with saturated *d_S_* values)

The least-squares algorithm as implemented in the Kitsch programme in PHYLIP tries to derive rooted phylogenies whose branch lengths are least different from the supplied distance matrix. In other words, the following expression is minimized:

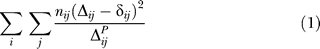
where the term Δ*_ij_* is the codeml-estimated *d_S_* distance between two sequences, and δ*_ij_* is the corresponding distance on the derived tree. The classical Fitch-Margoliash method [59] uses 2.0 for the term *P,* thus assuming that the variance is proportional to the square of the measured distances (*d_S_*), or that distance measurement errors are proportional to the expected value of the distance. However, our simulations show that errors in measuring *d_S_* grow more rapidly as synonymous sites approach saturation. We therefore used a *p* of 3.0 to down-weight less reliable large *d_S_* values. Missing values from the matrix that usually corresponded to saturated *d_S_* were ignored by assigning a weighting of zero: *n_ij_* = 0. Otherwise, *n_ij_* was 1.

We further modified the algorithm to avoid pernicious “long-branch attraction” errors due specifically to missing values. Pathological tree topologies containing branch joins based only on missing values were down-weighted by assigning an additional large weighting factor (of 10,000) to each occurrence. Given that we selected our initial sets of sequences using hierarchical single-linkage clustering, there can never be subgroups without any *d_S_* relationships between them. Trees with pathological joins can never be the globally optimal solution. The additional weighting term allows optima to be found away from these gaps in the optimisation landscape.

We used 50 random tree topologies as well as the hierarchical single-linkage cluster as initial starting points for branch and bound search. To avoid redundant searches, a lookup table was used to associate calculated tree scores with the MD5 128-bit hashes of each normalised topology. This greatly speeded up the algorithm and allowed wider ranging analyses of larger tree branches using more modest computing resources.

### Inferring orthology by congruence with the species tree

Orthology and paralogy relationships among the transcripts were inferred automatically by minimising the number of duplications that must be invoked to reconcile the transcript phylogeny with the species tree [60]. In this study, we were only applying the orthology prediction pipeline to sequences from two species, and because the Kitsch programme produces rooted bifurcating trees, the algorithm of Zmasek and Eddy [60] can be greatly simplified. Any node whose two branches each contain only dog sequences and human sequences, respectively, can be mapped to the last common ancestor of the dog and human on the species tree. All dog and human sequences in the clade defined by such a node represent descendants of a single original gene and are hence orthologues (e.g., clades 1 and 2 in Figure 3C). If the clade contains one dog and many human genes, a one-to-many relationship can be inferred. One-to-one, many-to-one, and many-to-many dog-to-human relationships were assigned in a similar manner. The remaining dog and human transcripts represent “orphaned” genes whose corresponding orthologue in the other species has been lost (e.g., H_3A_ and H_3B_ in Figure 3C). Orphans result from either (true) lineage-specific deletions (including conversions to a pseudogene) or gene prediction failures.

### Choosing representative transcripts

For each gene, we chose a single representative transcript from the phylogenies by applying four heuristics: first, we ruled out all orphaned transcripts outside of orthologue clades (Figure 2B, step 7). Second, for genes with multiple transcripts in the same orthologue clade, we chose progressively transcript pairs deriving from both species with the smallest *d_S_* to each other (Figure 2B, step 8). Third, where there was a set of orthologue clades of transcripts which map onto an identical complement of genes (e.g., clades 1 and 2 in Figure 3C each contain transcripts from the genes D_1_, H_1_, and H_2_), then these genes are orthologues of each other and their representative transcripts were chosen from the orthologue clade with the smallest branch lengths. (The branch length of clade 1 in Figure 3C, represented by a grey arrow, is shorter than that for clade 2.) Fourth, for genes with transcripts in different orthologue clades, the representative transcript with the smallest *d_S_* to transcripts from the other species in the clade was chosen (e.g., H_2A_ is chosen over H_1B_ in Figure 3D).

Genes whose representative transcripts are from the same clade were paralogous to each other if they were from the same species, and orthologous if they were from different species.

### Separating merged genes

To recover physically adjacent genes that have been mispredicted as a single merged gene, we searched for genes with multiple nonoverlapping transcripts. As described above, we only considered transcripts in orthologue clades. Thus, considering the canine gene with ten transcripts in Figure 4, transcripts 5–8 were not found in orthologue clades and would have been discarded first. The representative transcript can be identified by the procedures described above (transcript 2). Any transcripts from the same gene with overlapping Ensembl genomic coordinates were then eliminated (transcript 9). The remaining nonoverlapping transcripts (transcripts 1, 3, 4, and 10) represent one or more distinct genes. A representative transcript (transcript 1) could then be identified in turn for this newly separated gene. This procedure was applied recursively until all apparently merged genes (transcripts 1–4) were separated.

### Conserved syntenic gene order

**Figure 10:**
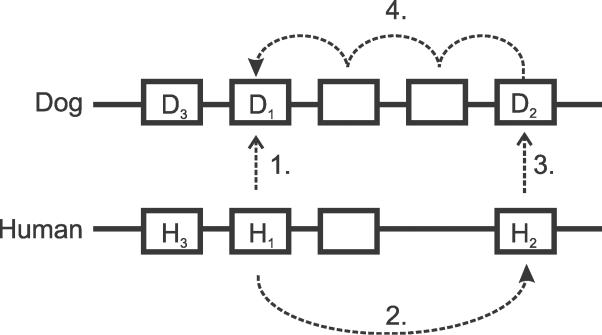
Calculating Minimum Syntenic Distance for Orthologues The minimum syntenic distance is the smallest difference in gene order between neighbours of its orthologues in the other species. Starting from human gene H_1_, the chromosomal location of its dog orthologue D_1_ is noted (step 1). The flanking genes (within a window of 20 sets of orthologues) are searched for the nearest neighbouring human gene with an orthologue on the same chromosome as D_1_. Thus, the immediate neighbour to the right of H_1_ can be ignored because it does not have an orthologue on the same chromosome as D_1_ (step 2). The subsequent gene H_2_ has a dog orthologue (step 3) D_2_ on the same chromosome as D_1_. The syntenic distance for gene H_1_ in the downstream direction is calculated to be four genes, by counting the number of intervening genes (using Ensembl gene loci) between D_1_ and D_2_ (step 4). Upstream of H_1_ and D_1_, however, no genes have been inserted after the next orthologous genes H_3_ and D_3_. The minimum syntenic distance for H_1_ is thus 1.

If gene order was conserved, adjacent orthologues in one species should be neighbours in the other. In many cases, contiguity appeared to have been interrupted by gene insertions in one species (or corresponding losses in the other). We calculated the minimum syntenic distance for a gene as the smallest difference in gene order between neighbours of its orthologues in the other species. This process is illustrated by the example in Figure 10.

### Pseudogenes

Likely pseudogenes were identified by the presence of short introns (less than 10 bp), indicating frameshift or in-frame stop codon disruptions, or by the lack of conserved syntenic gene order in dispersed genes (syntenic distance > 20 genes). We conservatively labelled as a pseudogene any 1) dispersed gene with one or more disruptions, 2) syntenic gene with multiple disruptions, and 3) dispersed single exonic gene. The latter represent mostly retrotransposed pseudogenes (see Results).

Some orthologous clades had so many apparent pseudogenes (there were three with more than 70 genes each) spread across the genome that many had an orthologue with a syntenic distance of <20 genes simply by chance. To identify these pseudogenes, we defined widely scattered families as those with members on more than four chromosomes. True orthologues were determined using the criterion of three or more exons with matching exon boundaries in both species. All the members of the orthologue clade with two or fewer exons with nonmatching exon boundaries were labelled as pseudogenes.

### Orthologous chromosomal segments

“Micro-syntenic” blocks of orthologous genes were constructed by grouping together successive genes with conserved gene order and orientation among predicted orthologues in the other species. “Macro-syntenic” blocks were created by concatenating contiguous micro-syntenic blocks that, after rearrangements and inversions, corresponded to a single block of orthologues with conserved gene order in the other species [6].

For orthologous genes in “many” (e.g., one-to-many) relationships, any of the alternative orthologues that conserve gene order and strand qualify the gene for inclusion in a micro-syntenic block. Both types of syntenic blocks are thus directional and species-specific.

### Ensembl and Inparanoid orthology prediction

Ensembl orthologue predictions were obtained from the Compara database (version 27.1). Orthologue sets were created by joining together pairwise orthology predictions using single-linkage clustering. Ensembl orthologues were based on the longest transcripts of each gene [11,14], and where alignment and *d_S_* data are given, we have calculated these from the corresponding sequences.

Inparanoid version 1.35 was used to predict orthology from BLASTALL alignments results (National Center for Biotechnology Information [NCBI] version 2.2.12) for the longest gene transcripts, as described previously [61]. We used the BLOSUM80 matrix and an X drop-off value of 150.

### Conservation of exon boundaries

The exon loci for each gene were obtained from Ensembl and mapped onto peptide sequences. We identified conserved exon boundaries if they fell within three corresponding codon positions of each other. We ignored exons that fell either outside or at the two ends (distal three codons) of the aligned regions. We also overlooked cases where a single intron was missing in one sequence if the pair included three or more otherwise aligned exons.

### GO terms

GO [62] assignments for all human genes (EnsMart version 27.1) were retrieved from Ensembl. GO terms for dog genes were assigned on the basis of their orthology relationships with human genes. To summarise the large number of overlapping GO terms in Table 3, we used only terms from the overarching GO-SLIM set [63]. This consists of 36 component, 41 function, and 52 biological process terms. The statistical significance of overrepresentations of each GO term among human- and dog-specific paralogues was evaluated using the cumulative hypergeometric distribution with reference to the representation of that term among all orthologues. Only GO terms that are significantly overrepresented (*p* < 10^−4^) among pseudogenes and in-paralogues are presented in Tables 3 and 8.

## Abbreviations

GO: gene ontology
KRAB-ZnF: KRAB-zinc finger
PhyOP: phylogenetic orthology and paralogy
